# A Mobile Phone–Based Life-Skills Training Program for Substance Use Prevention Among Adolescents: Cluster-Randomized Controlled Trial

**DOI:** 10.2196/26951

**Published:** 2021-07-13

**Authors:** Severin Haug, Raquel Paz Castro, Andreas Wenger, Michael Patrick Schaub

**Affiliations:** 1 Swiss Research Institute for Public Health and Addiction at Zurich University Zurich Switzerland

**Keywords:** life skills, substance use, prevention, adolescents, mobile phone

## Abstract

**Background:**

Life skills are abilities for adaptive and positive behavior that enable individuals to deal effectively with the demands and challenges of everyday life. Life-skills training programs conducted within the school curriculum are effective in preventing the onset and escalation of substance use among adolescents. However, their dissemination is impeded due to their large resource requirements. Life-skills training provided via mobile phones may provide a more economic and scalable approach.

**Objective:**

The goal of this study was to test the appropriateness (ie, acceptance, use, and evaluation) and short-term efficacy of a mobile phone–based life-skills training program to prevent substance use among adolescents within a controlled trial.

**Methods:**

The study design was a two-arm, parallel-group, cluster-randomized controlled trial with assessments at baseline and follow-up assessments after 6 and 18 months. This report includes outcomes measured up to the 6-month follow-up. The efficacy of the intervention was tested in comparison to an assessment-only control group. The automated intervention program *SmartCoach* included online feedback and individually tailored text messages provided over 22 weeks. The contents were based on social cognitive theory and addressed self-management skills, social skills, and substance use resistance skills. Linear mixed models and generalized linear mixed models, as well as logistic or linear regressions, were used to investigate changes between baseline and 6-month follow-up in the following outcomes: 30-day prevalence rates of problem drinking, tobacco use, and cannabis use as well as quantity of alcohol use, quantity of cigarettes smoked, cannabis use days, perceived stress, well-being, and social skills.

**Results:**

A total of 1759 students from 89 Swiss secondary and upper secondary school classes were invited to participate in the study. Of these, 1473 (83.7%) students participated in the study; the mean age was 15.4 years (SD 1.0) and 55.2% (813/1473) were female. Follow-up assessments at 6 months were completed by 1233 (83.7%) study participants. On average, program participants responded to half (23.6 out of 50) of the prompted activities. Program evaluations underlined its appropriateness for the target group of secondary school students, with the majority rating the program as helpful and individually tailored. The results concerning the initial effectiveness of this program based on 6-month follow-up data are promising, with three of nine outcomes of the intention-to-treat analyses showing beneficial developments of statistical significance (ie, quantity of alcohol use, quantity of tobacco use, and perceived stress; *P*<.05) and another three outcomes (ie, problem drinking prevalence, cannabis use days, and social skills) showing beneficial developments of borderline significance (*P*<.10).

**Conclusions:**

The results showed good acceptance of this intervention program that could be easily and economically implemented in school classes. Initial results on program efficacy indicate that it might be effective in both preventing or reducing substance use and fostering life skills; however, data from the final 18-month follow-up assessments will be more conclusive.

**Trial Registration:**

ISRCTN Registry ISRCTN41347061; https://doi.org/10.1186/ISRCTN41347061

## Introduction

During adolescence numerous biological, psychological, and social transitions take place, which determine a young person’s development and future [1,2]. These transitions allow adolescents to develop skills in order to achieve greater autonomy, build relationships with peers, develop a positive body image, and find one’s identity. However, they are also accompanied by an increased willingness to take risks during a time when the cognitive functions of the brain (eg, to regulate emotions) are not yet fully developed [3]. Shifts in emotional regulation as well as increased risk behavior increase the susceptibility of an individual to develop mental and substance use disorders. These disorders are largely responsible for the health burden of 10- to 24-year-old individuals [4]. Substance use, as well as the development of substance use disorders, co-occur with mental disorders and typically first arise throughout the adolescent years [1].

The prevalence of lifetime and recent alcohol and tobacco use increases sharply in both genders from 11 to 15 years of age [5]. In Switzerland, the lifetime prevalence of alcohol use increased from 22% among 11-year-old boys to 70% among 15-year-old boys, and from 11% among 11-year-old girls to 69% among 15-year-old girls [6]. The proportion of pupils who reported having smoked cigarettes at least once in their life increased from 6% among 11-year-old boys to 35% among 15-year-old boys, and from 2% among 11-year-old girls to 30% among 15-year-old girls. This age range reflects a critical time when substance prevention programs should be implemented.

A systematic review of studies, which examined the efficacy of prevention, early intervention, and harm reduction in adolescents for tobacco, alcohol, and illicit drugs, illustrated the effectiveness of taxation, public consumption bans, advertising restrictions, and minimum legal age. Additionally, promising effectiveness of preventive interventions, which provide life-skills training in educational settings, was shown [7]. Using schools as a medium to reach adolescents with preventive interventions is particularly suitable, as it facilitates the delivery and access to adolescents within compulsory secondary education. A Cochrane review on school-based programs for the prevention of tobacco smoking demonstrated a significant intervention effect from the combination of social competence and social influence interventions [8]. Another Cochrane review on school-based prevention programs for alcohol misuse among young people concluded that certain generic psychosocial and developmental prevention programs can be effective [9]. A large proportion of generic programs tending to social competencies and social influences, which were referenced in the reviews mentioned above, are defined as life-skills training and are based on Bandura’s social learning theory [10]. This theory explains that children and adolescents discover substance use by modeling, imitation, and reinforcement, which is influenced by individual cognitions and attitudes. Moreover, in light of the social influences approach [11], substance use susceptibility increases as a result of a lack of personal and social skills, and adolescents begin drug use because of pressure from friends, family, and the media. According to the definition from the World Health Organization, life skills are “abilities for adaptive and positive behavior that enable individuals to deal effectively with the demands and challenges of everyday life” [11].

Life-skills intervention programs to prevent substance use [12-14] primarily combine training in self-management skills (eg, adapting to stress, emotional self-regulation, and goal setting), social skills (eg, assertiveness and communication skills), and skills facilitating the resistance to substance use (eg, opposing peer pressure to drink alcohol, identifying and resisting media influences that promote cigarette smoking, and correcting normative misperceptions of substance use). In spite of the fact that these life-skills training programs were compelling at preventing the onset [8,14,15] of using an explicit substance or at reducing problematic substance use [9], their implementation and dispersal in schools present genuine difficulties [16]: teachers or other professionals need time, training, knowledge, and skills to prepare and administer such programs [17].

Digital interventions have great potential to overcome the above-mentioned obstacles that hinder successful program implementation and larger-scale dissemination of life-skills training in schools. These programs have a large reach at low cost and offer the ability to deliver uniquely personalized content automatically, which can be accessed anytime and anywhere. Furthermore, digital interventions might be more appealing for adolescents because they can better ensure privacy and tailor contents to their needs. A systematic review of digital alcohol and other drug prevention programs [18] identified nine trials, six of which demonstrated significant, but modest, effects for alcohol and/or other drug use outcomes. The programs were delivered in the United States, Australia, and the Netherlands and provided between 1 and 12 online curriculum-based standard lessons or tailored feedback. All programs were universal (ie, delivered interventions to all students regardless of their level of risk) and were primarily based on principles of the social learning theory [10], the social influences approach [19], and the social cognitive theory [20,21].

A promising way of delivering preventive services, besides conventional personal computers, is to do so remotely by using mobile technologies. Almost all (99%) adolescents between the ages of 12 and 19 years in Switzerland, as in most other developed countries, own a mobile phone. Compared to services that can only be accessed at particular times or places, they provide a targeted and confidential means of intervention delivery [22]. Mobile phone–based interventions can provide almost constant support to users, in comparison to interventions that can only be accessed at specific times or locations, and they provide a discrete and confidential means of intervention delivery [23]. Mobile phone text messaging, in particular, is a suitable means of delivering individually tailored messages via mobile phones. This interactive service allows cost-effective, instantaneous, direct delivery of messages to individuals. Recent reviews underline the potential efficacy of text messaging–based interventions to reduce alcohol and tobacco use for different at-risk target groups, including adolescents and young adults [24,25].

*Ready4life* is a mobile phone–based life-skills training program for substance use prevention. Its acceptance and potential effectiveness was tested within a pre-post study in Switzerland [26]. Program participants were vocational school students with a mean age of 17 years, who received up to three weekly text messages over 6 months. The *ready4life* program was based on social cognitive theory and addressed self-management skills, social skills, and substance use resistance skills. Active program engagement was encouraged through interactive features, such as quiz questions, message and picture contests, and integration of a friendly competition with prizes, in which program users collected credits with each interaction. A total of 4 out of 5 eligible students participated in the program and the associated study. Pre-post comparisons, between baseline and follow-up assessments, revealed decreased perceived stress and increases in several life skills that were addressed. The proportion of adolescents with at-risk alcohol use significantly declined from 20% at baseline to 16% at follow-up.

Based on these promising findings [26], a similar universal prevention program was developed for the target group of secondary school students; these students are slightly younger than vocational school students and their substance use is not yet fully advanced [5]. Our main hypothesis concerning the final follow-up at month 18 is that the individually tailored intervention program will be more effective than assessment only in preventing the onset and escalation of problematic alcohol and tobacco use.

This study presents (1) the results on appropriateness (acceptance, use, and evaluation of duration, intensity, tailoring, helpfulness, comprehensibility, etc) of this program as well as (2) initial results on its efficacy considering 6-month follow-up assessments of this controlled trial.

## Methods

### Objectives and Study Design

This study aimed at testing the acceptance and short-term efficacy of *SmartCoach*, a mobile phone–based life-skills training program to prevent substance use among secondary school students. The efficacy of the intervention was tested in comparison to an assessment-only control group, considering data from the first follow-up assessment after 6 months.

### Participants, Setting, and Procedure

We tested the intervention program in secondary and upper secondary school students, typically aged between 14 and 17 years. Secondary schools in the German-speaking part of Switzerland were invited to participate in the study by cooperating regional centers for addiction prevention. Employees of these centers arranged 60-minute information sessions in participating secondary school classes during regular school lessons reserved for health education. These information sessions were led by junior scientists from the Swiss Research Institute for Public Health and Addiction, who were experienced in work with young people, experienced in the provision of preventive interventions, and trained on the study and the program to be delivered.

The parents of secondary school students below the age of 16 years were informed at least one week in advance of this session. They received a letter including information about the study and the intervention program and were asked to give written informed consent regarding their child’s participation in the study. Adolescents aged 16 years or older gave their own informed consent.

Within the first half of the information sessions in the school classes, the junior scientists raised awareness about the importance of life skills to effectively cope with the demands and challenges of everyday life. For this purpose, they used video sequences demonstrating typical stressors and demands for this age group (eg, search for an apprenticeship, exam stress, and peer pressure for substance use) and different strategies to cope with them. The importance of emotional regulation skills and social skills to effectively cope with these stressors were discussed based on case vignettes. Subsequently, the students were informed about, and invited to participate in, a study testing innovative channels for the provision of life-skills training. To ensure adherence to the study protocol and representativeness of the sample [27], a reward of CHF 10 (US $10.90) for participation in each of the two follow-up assessments was announced.

Students were invited to participate in the study if they met the following criteria: (1) were a minimum age of 14 years, (2) owned a mobile phone, and (3) provided parental informed consent if they were under 15 years of age. Informed consent was obtained online from all study participants. Subsequently, they were invited to choose a username, provide their mobile phone number, and fill in the baseline assessment directly on their mobile phone.

Participants of the intervention group received additional questions, which were necessary for the tailoring of the intervention content. Furthermore, for participants of the intervention group, the mobile phone–based intervention program and its association with a friendly competition was described in detail. Subsequently, participants of the intervention group received individually tailored web-based feedback directly on their mobile phone (see Intervention Program section). During the subsequent 6 months, participants of the intervention group received individually tailored mobile phone–based life-skills training.

Participants of the assessment-only control group were thanked for their study participation and were informed about their group assignment and their reward for participation in the follow-up assessment.

Follow-up assessments in both study groups were conducted using a similar procedure: participants were invited to the online follow-up assessments via SMS text messaging, which included a link to the follow-up survey. Nonresponders were additionally addressed via computer-assisted telephone interviews conducted by research assistants. Study participants were recruited between March 2019 and March 2020. The 6-month follow-up assessments were conducted between August 2019 and September 2020.

### Ethical Review and Trial Registration

The study protocol was approved on June 21, 2018, by the Ethics Committee of the Faculty of Arts and Sciences at the University of Zurich, Switzerland (approval No. 18.6.5). The trial was executed in compliance with the Helsinki Declaration. The trial was registered on July 21, 2018, at the ISRCTN Registry (ISRCTN41347061).

### Randomization and Allocation Concealment

To avoid spillover effects within school classes, we conducted a cluster-randomized controlled trial using a school class as a randomization unit. Due to the heterogeneity of students in the different secondary schools, we used a separate randomization list for each school (ie, stratified randomization). Furthermore, to approximate equality of sample sizes in the study groups, we used block randomization with computer-generated randomly permuted blocks of 4 cases [28].

Junior scientists supervising the baseline assessment were blinded to the group allocation of school classes. In addition, group allocation was not revealed to participants until they had provided their informed consent, username, mobile phone number, and baseline data. Furthermore, the research assistants who performed the computer-assisted follow-up assessments for primary and secondary outcomes were blinded to the group allocation.

### Intervention Program

#### Theoretical Background and Intervention Contents

The intervention elements of the program were based on social cognitive theory [20,21]. The key concepts of this theory, which were addressed within *SmartCoach*, were (1) outcome expectations, (2) self-efficacy, (3) observational learning, (4) facilitation, and (5) self-regulation. Their implementation within the *SmartCoach* program is described in more detail within the study protocol [29].

#### Technological Background

The intervention program was developed using the MobileCoach system. Technical details of the system are described elsewhere [30,31]. The MobileCoach system is available as an open-source project. Password protection and Secure Sockets Layer encoding were used to ensure the privacy and safety of data transfer.

#### Individually Tailored Feedback

Individually tailored web-based feedback was provided to study participants of the intervention group immediately after completion of the online baseline assessment within the school classes. It comprised seven screens, including textual and graphical feedback on stress in general, individual level of stress in various domains, individual applied and suggested coping strategies, as well as individual level of social skills. Instruments for the assessment of stress and coping strategies were derived from the Juvenir 4.0 study, a national survey on stress in adolescents with more than 1500 participants [32]. Data from this survey were also used to provide age- and gender-specific feedback on individual stress level.

#### Text Messages

For a period of 22 weeks, program participants received between two and four individualized text messages per week on their mobile phone. These messages were generated and sent by the fully automated system. Within the first 7 weeks, the messages focused on self-management skills (eg, coping with stress, emotional self-regulation, or management of feelings of anger and frustration). In weeks 8 to 17, the messages focused on social skills (eg, making requests, refusing unreasonable requests, and meeting new people). In weeks 18 to 22, the text messages focused on substance use resistance skills (eg, recognizing and resisting media influences, correcting normative misperceptions of substance use, or understanding the associations of self-management skills and social skills with substance use). The messages were tailored according to the individual data from the baseline assessment and were based on text messaging assessments during the program runtime (eg, on substance use or on the individual’s emotional state).

Several interactive features, such as quiz questions, tasks to create individually tailored if-then behavior plans based on implementation intentions, and message contests, were implemented within the program. Due to the wide dissemination of smartphones among adolescents [22], several messages also included hyperlinks to audio files (eg, audio testimonials and motivational podcasts) as well as to thematically appropriate video clips, pictures, and related websites. Table 1 displays the sequence and content of the text messages.

**Figure 1:**
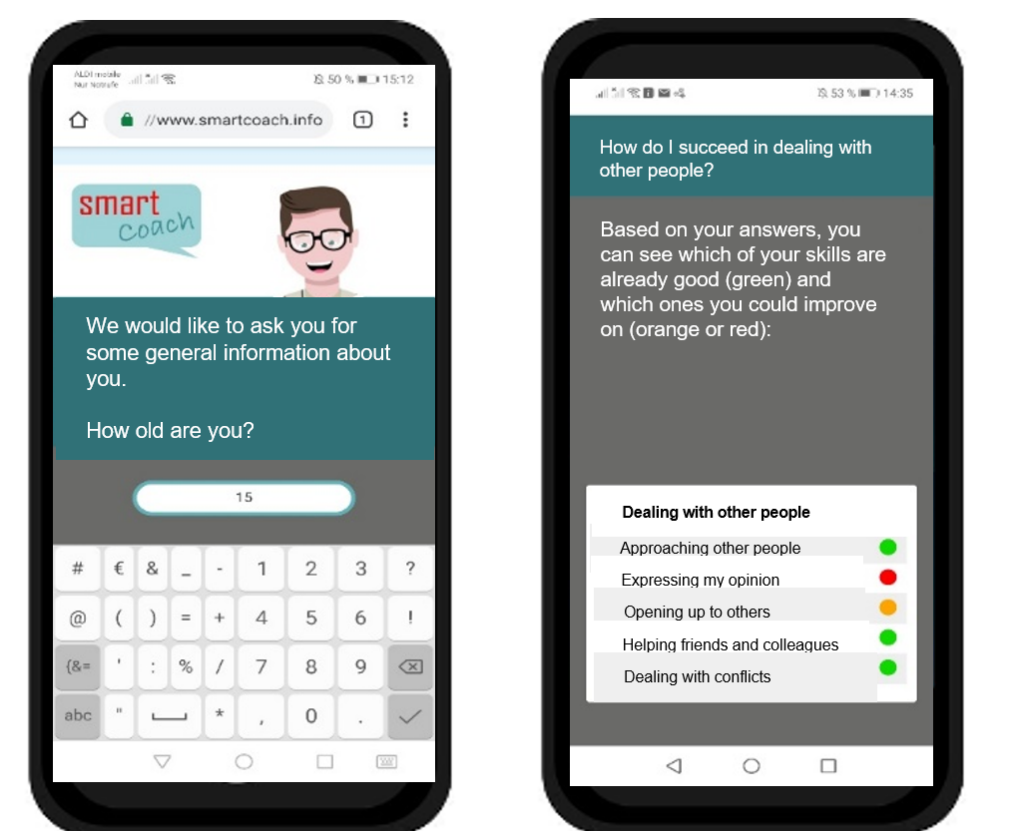
Screenshots (translated into English) from the *SmartCoach* program: baseline assessment (left) and feedback on social skills (right).

**Figure 2:**
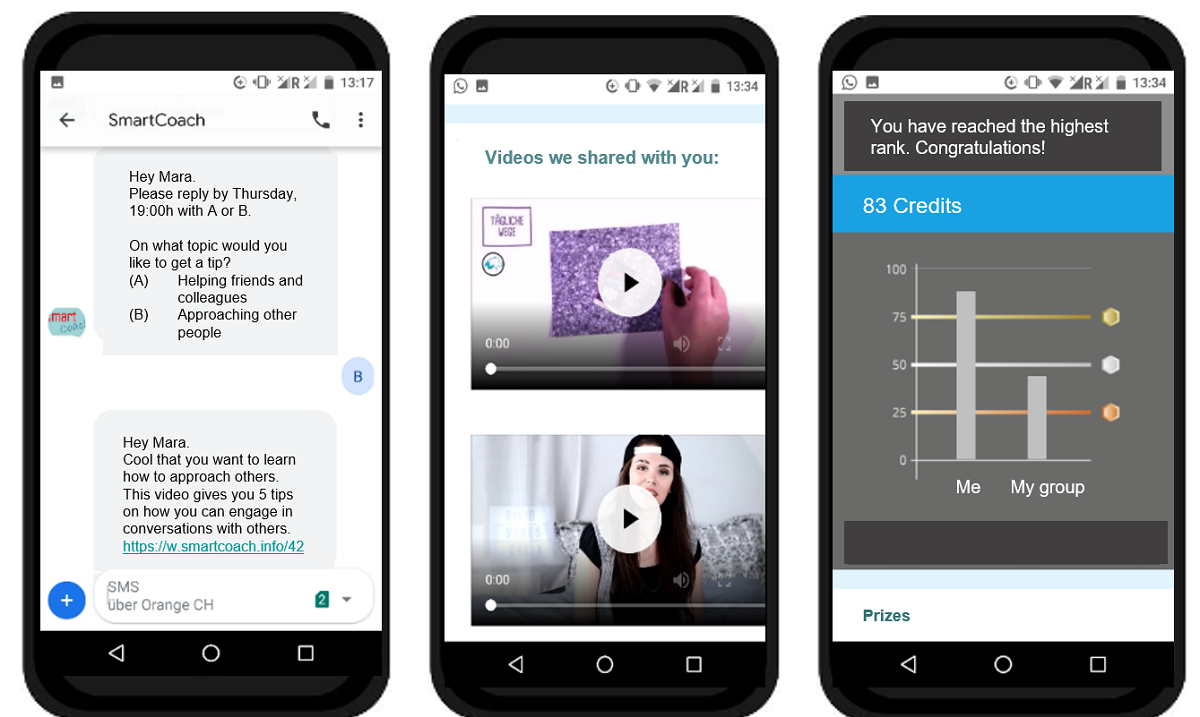
Screenshots (translated into English) from the *SmartCoach* program: text messages (left), video clips (middle), and friendly competition (right).

Figures 1 and 2 show a selection of intervention elements from the *SmartCoach* program.

**Table 1:** Sequence and content of text messages within the *SmartCoach* program.

Week No.	Content	Required activities
1	Introduction to self-management skills Origin and function of stress	Reply to quiz question Click on video link
2	Quiz on common stressors	Reply to quiz question Click on video link
3	Tailored stress reduction strategies for individual stressors	Reply to text message with options Click on video or website link
4	Self-challenge on general stress reduction strategies	Reply to text message with options Reply to text message on successful application of chosen strategy
5	Quiz on eustress versus distress	Reply to quiz question Click on video link
6	Tailored stress reduction strategies for individual stressors	Reply to text message with options Click on video or website link
7	Group contest on preferred stress management strategy	Post a picture and text message on individually preferred strategy Voting of others’ posts Viewing of most-voted posts
8	Introduction to socials skills Quiz on social skills	Click on link to an overview picture Reply to quiz question Click on link to picture
9	Tailored strategies for improving personal social skills	Reply to text message with options Click on video or website link
10	Quiz on use of body language in different situations	Reply to quiz question Click on video link
11	Tailored strategies for improving personal social skills	Reply to text message with options Click on video or website link
12	Self-challenge on strategies to improve social skills in different areas	Reply to text message with options Reply to text message on successful application of chosen strategy
13	Origin of smartphone addiction	Reply to quiz question Click on video link
14	Quiz on associations between smartphone use and stress, tailored to gender	Reply to quiz question Click on video links
15	Self-challenge on smartphone detox	Reply to text message with options Reply to text message on successful detox in chosen situation Click on video link
16	Quiz on recognition of peer pressure	Click on link to the first part of the video Reply to quiz question Click on link to the second part of the video
17	Group contest on favorite social situation	Post a picture and text message on favorite social situation Voting of others’ posts Viewing of others’ posts
18	Introduction to substance use resistance skills Quiz on substance use prevalence (alcohol and tobacco) in reference group and normative feedback	Click on link to an overview picture Reply to quiz question
19	Quiz on the presence of tobacco advertisements directed to adolescents in everyday life	Reply to quiz question Click on video link
20	Quiz on risks of alcohol use	Reply to quiz question Click on website link
21	Tailored information on social consequences of alcohol use	Click on video link
22	Group contest on motivation for abstinence or low-risk alcohol use	Post a motivational text message Voting of others’ posts Viewing of others’ posts

#### Prize Draw

To stimulate active program engagement, program use was associated with a friendly competition, which allowed program users to collect credits for each interaction (eg, answering monitoring text messages, participating in quizzes, creating messages or pictures within contests, and accessing video links integrated in text messages). The more credits participants collected, the higher their chances of winning one of 10 prizes, which were part of a prize draw (10 cash prizes of CHF 50 [US $54.50] each) after program completion. Participants were able to compare their number of credits with that of other program participants in their group (ie, similar starting date) at any time from an individual profile page. As can be seen in Figure 2 on the right, this was a mixture of feedback on the absolute number of credits for a participant and the relative score compared to the average number of credits for the reference group. However, the absolute individual score was ultimately decisive for the chances of winning a prize, so a win was possible when reaching the bronze level (25 credits), the chance was twice as high when reaching the silver level (50 credits), and there was a three-fold chance of winning when reaching the gold level (75 credits).

### Assessments and Outcomes

#### Demographics

At baseline, demographic variables (ie, age, sex, and immigration background) as well as type of school (ie, secondary or upper secondary school) were assessed.

#### Program Use and Evaluation

To obtain the number of program participants who unsubscribed from the program within the program runtime of 6 months, we analyzed the log files of the MobileCoach system, in which all incoming and outgoing text messages were recorded. Using these log files, we also assessed the number of activities performed (eg, replies to text messaging prompts, accessing weblinks within text messages, and participating in contests) during the program. At follow-up, we assessed another aspect of SMS usage by asking the participants whether they usually (1) read through the text messages thoroughly, (2) took only a short look at them, or (3) did not read the text messages.

Furthermore, we evaluated whether (1) the number of received text messages was felt to be appropriate, (2) the duration of the program was adequate, (3) the participants would recommend the program to others, (4) the text messages were comprehensible, (5) the text messages were helpful, and (6) the text messages were perceived as individually tailored. Finally, program participants were asked to rate the program and different program elements, using the response categories *very good*, *good*, *less than good*, *bad*, and *don’t know*.

#### Outcomes

Baseline and follow-up assessments included the following:

1. Problem drinking and alcohol use in the preceding 30 days, assessed by the short form of the Alcohol Use Disorders Identification Test–Consumption Items (AUDIT-C) [33]. This test is comprised of three items: (1) frequency of alcohol consumption, (2) quantity of alcohol consumption, and (3) binge drinking. Pictures were used to illustrate the quantity of a standard drink, which corresponded to 12 g to 14 g of pure alcohol. Based on a validation study of a large German sample, a cutoff score of ≥5 was used [34].
2. The 30-day point prevalence rate for smoking abstinence (ie, *not having smoked a puff* within the past 30 days according to the criteria of the Society for Nicotine and Tobacco Research [35]).
3. Quantity of cigarettes smoked in the preceding 30 days, assessing by the number of smoking days and the typical number of cigarettes smoked per smoking day.
4. Cannabis use in the preceding 30 days, assessed by an item of the HBSC (Health Behaviour in School-aged Children) study [36] addressing the number of cannabis consumption days.
5. Perceived stress, assessed by a single item from the Swiss Juvenir study [32]—“How often have you had the feeling of being overstressed or overwhelmed in the last month?”—with answer options ranging from 1 (never) to 5 (all the time).
6. Well-being, assessed by the 5-item World Health Organization Well-Being Index (WHO-5) [37], with the final score ranging from 0, representing the worst imaginable well-being, to 100, representing the best imaginable well-being.
7. Social skills, assessed by the brief version of the 10-item Interpersonal Competence Questionnaire (ICQ-10) [38] addressing the following domains: (1) initiation of relationships, (2) negative assertion, (3) disclosure of personal information, (4) emotional support, and (5) conflict management.

The primary outcomes, according to the study protocol [29], are (1) prevalence of problem drinking in the preceding 30 days according to the AUDIT-C and (2) prevalence of cigarette smoking in the preceding 30 days (ie, having smoked at least a puff, according to the criteria of the Society for Nicotine and Tobacco Research [35]). Secondary outcomes were (1) prevalence of cannabis use in the preceding 30 days (ie, having used cannabis at least once), (2) quantity of alcohol use in the preceding 30 days, (3) quantity of cigarettes smoked in the previous 30 days, (4) frequency of cannabis use in the preceding 30 days, (5) perceived stress, (6) well-being, and (7) social skills.

### Data Analyses

Descriptive statistics were used to present indicators of program use and evaluation. In order to examine baseline differences between participants of the intervention and control groups, we performed chi-square tests for categorical variables as well as *t* tests and Mann-Whitney *U* tests for continuous variables. The same tests were applied to assess whether participants who were lost to follow-up differed from those who responded, as a function of the study group.

We analyzed data according to the intention-to-treat (ITT) principle. For the ITT analyses, we used multiple imputation procedures as described elsewhere [39]. We imputed for each group separately to preserve homogeneity within the groups and potential interventional effects. Overall predictors of missing data at follow-up were gender, immigration background, education, and number of students within a school class. Differential predictors of missing data at follow-up were problem drinking, tobacco smoking, and use of the program. Thus, these predictors were part of all imputation models for the study’s primary and secondary outcomes. The remaining study outcome predictors were variables that correlated at least weakly with these (*r*>0.20). Binary variables were imputed using logistic regression, categorical variables using multinomial logit models, and continuous variables using predictive mean matching. We examined 50 data sets and no systematic bias in convergence was revealed; thus, the final inferences were derived from this solution.

Next, we calculated the intraclass correlation (ICC) for primary and secondary outcomes. In our study, the ICC determines the extent to which study outcomes vary across classrooms. If an ICC is close to 0, standard regressions provide unbiased coefficients, whereas an ICC higher than 0 indicates that hierarchical models are needed to avoid a type I statistical error. In previous studies, ICCs between 0.05 and 0.10 were considered negligible [40,41]. However, it is an open debate as to how well the ICC performs depending on the underlying data [42]. Thus, we opted for a conservative approach and conducted linear mixed models (LMMs) and generalized linear mixed models (GLMMs) where the ICC was higher than 5%, and logistic or linear regressions where the ICC was below 5%.

Within LMMs and GLMMs, we modeled a random intercept for school class, while predictors and covariates were identical to logistic or linear regressions. Analyses of binary outcomes focused on follow-up values. Independent variables included baseline values for the binary variables of interest, group as a predictor, and variables for which baseline differences were observed as covariates. Analyses of continuous outcomes included change in score from baseline to follow-up as the dependent variable. Independent variables included group as a predictor and variables for which baseline differences were observed. We included in all models a covariate that modeled the possible effect of the lockdown measures undertaken in Switzerland between February 28 and June 22, 2020, because of the COVID-19 pandemic. During this period, several parts of students’ lives were affected (eg, schools and/or bars were closed), which may have had an effect on our outcomes. The results from the imputed data set were cross-checked with the nonimputed data set. Results with a type I error rate of *P*<.05 on two-sided tests were considered statistically significant. Analyses were performed using SPSS, version 25 (IBM Corp), and R, version 3.6.1 (The R Foundation). Multiple imputation was conducted with the mice (multivariate imputation by chained equations) package in R [43], and LMM and GLMM were conducted with the lme4 (linear mixed-effects 4) package in R [44].

## Results

### Study Participants

**Figure 3:**
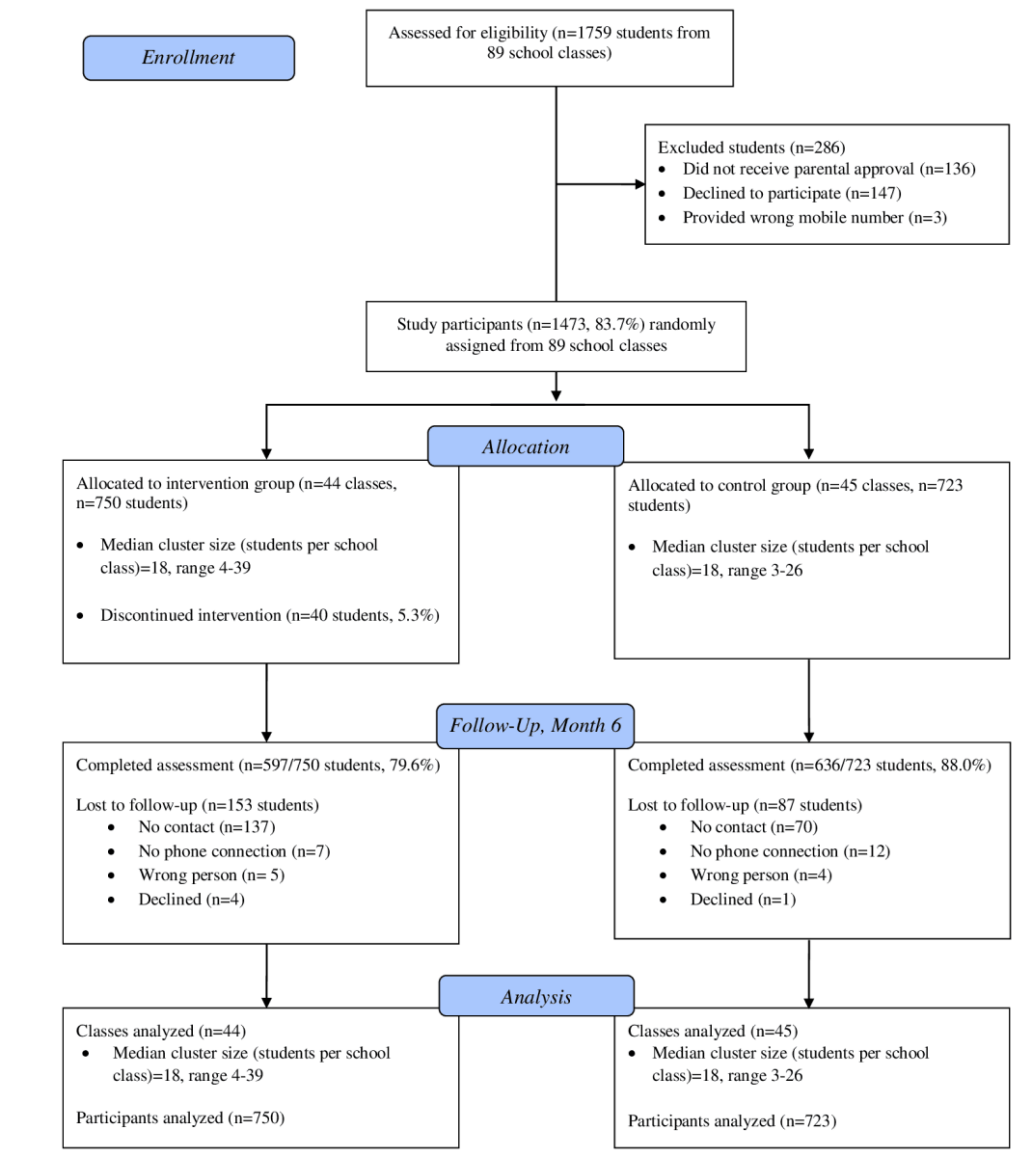
Participants’ progression through the trial.

Figure 3 depicts participants’ progression through the trial. At the online screening assessment, 1759 students were present in 89 classes. Of these, 1623 (92.3%) students received parental approval to participate, and 1473 (83.7%) students ultimately participated in the study. A total of 44 classes containing 750 students in total were randomly assigned to the intervention group, and 45 classes containing 723 students in total were assigned to the control group. Follow-up assessments at 6 months were completed by 597 out of 750 (79.6%) participants in the intervention group and 636 out of 723 (88.0%) participants in the control group.

Baseline characteristics for the study sample are shown in Table 2. The mean age was 15.4 years (SD 1.0), and 55.2% (813/1473) of the participants were female.

Baseline differences between the intervention and control groups were revealed for immigration background, education, prevalence of tobacco smoking and problem drinking, quantity of alcohol use, and perceived stress.

Concerning attrition bias, the analysis revealed that intervention group participants who were lost to follow-up reported more frequent tobacco use (Wald=6.38, *df*=1; *P*=.01) and problem drinking (Wald=8.97, *df*=1; *P*=.003) at baseline than controls.

**Table 2:** Baseline characteristics of the study sample.

Variable		Intervention group (n=750)	Control group (n=723)	Total (N=1473)	*P* value^a^	
**Sex, n (%)**						.34^b^
	Male	327 (43.6)	333 (46.1)	660 (44.8)		
	Female	423 (56.4)	390 (53.9)	813 (55.2)		
Age (years), mean (SD)		15.4 (1.0)	15.5 (1.0)	15.4 (1.0)	.052^c^	
**Immigration background, n (%)**						<.001^b^
	No immigration background	389 (51.9)	309 (42.7)	698 (47.4)		
	One parent born outside Switzerland	173 (23.1)	168 (23.2)	341 (23.2)		
	Both parents born outside Switzerland	188 (25.1)	246 (34.0)	434 (29.5)		
**Type of school, n (%)**						.007^b^
	Secondary school	165 (22.0)	203 (28.1)	368 (25.0)		
	Upper secondary school	585 (78.0)	520 (71.9)	1105 (75.0)		
**Tobacco smoking, preceding 30 days, n (%)**						.09^b^
	No	659 (87.9)	614 (84.9)	1273 (86.4)		
	Yes	91 (12.1)	109 (15.1)	200 (13.6)		
Quantity of cigarettes smoked, preceding 30 days, mean (SD)		5.3 (1.5)	7.9 (1.8)	6.6 (1.2)	.10^d^	
**Problem drinking, preceding 30 days, n (%)**						
	No	636 (84.8)	573 (79.3)	1209 (82.1)	.006^b^	
	Yes	114 (15.2)	150 (20.7)	264 (17.9)		
Quantity of alcohol use, preceding 30 days, mean (SD)		5.9 (16.3)	7.5 (16.4)	6.7 (16.4)	.003^d^	
**Cannabis use, preceding 30 days, n (%)**						.95^b^
	No	644 (85.9)	620 (85.8)	1264 (85.8)		
	Yes	106 (14.1)	103 (14.2)	209 (14.2)		
Cannabis use days, preceding 30 days, mean (SD)		0.77 (3.4)	0.78 (3.5)	0.78 (3.5)	.95^b^	
Perceived stress score^e^, mean (SD)		2.9 (0.9)	3.0 (0.9)	2.99 (0.9)	.04^c^	
Well-being score (WHO-5^f^), mean (SD)		52.9 (17.3)	51.6 (17.3)	52.3 (17.3)	.11^c^	
Social skills score (ICQ-10^g^), mean (SD)		14.9 (2.2)	14.9 (2.2)	14.9 (2.2)	.45^c^	

^a^*P* values for the comparison of the intervention and control groups.

^b^*P* value calculated from chi-square test.

^c^*P* value calculated from *t* test.

^d^*P* value calculated from Mann-Whitney *U* test.

^e^Perceived stress scores range from 1 (never) to 5 (all the time).

^f^WHO-5: 5-item World Health Organization Well-Being Index; final scores range from 0 (worst imaginable well-being) to 100 (best imaginable well-being).

^g^ICQ-10: 10-item Interpersonal Competence Questionnaire; final scores range from 5 (always poor/unable to handle social situations) to 20 (always good/able to handle social situations).

### Program Use and Evaluation

During the intervention program, which lasted for 22 weeks, 40 of the 750 (5.3%) program participants withdrew their participation. A total of 50 activities (eg, replying to text messaging prompts, accessing weblinks within text messages, and participating in contests) were prompted over the 6-month program. The mean number of activities carried out by participants was 23.6 (SD 15.9). A total of 9.6% (72/750) of participants did not take part in any of the activities prompted by the program. Low engagement with the program was established for 149 out of 750 (19.9%) students, who interacted with it only 1 to 10 times. A total of 14.0% (105/750) of the participants interacted 11 to 20 times, 13.3% (100/750) interacted 21 to 30 times, 24.9% (187/750) interacted 31 to 40 times, and 18.3% (137/750) interacted 41 to 50 times.

Of 597 participants with valid follow-up data, 563 (94.3%) answered the question regarding whether they had read the text messages. Of these, 70.9% (399/563) indicated that they *read the SMS messages thoroughly*, 27.0% (152/563) reported that they *took a quick look at the SMS messages*, and 1.6% (12/563) chose the predefined response category *I did not read the SMS messages*. The number of text messages received was rated as appropriate by 78.6% (442/562) of participants; 12.0% (90/562) would have preferred fewer messages, and 5.3% (30/562) would have preferred more text messages. Three-quarters of the participants rated the total length of the program as adequate (424/561, 75.6%); 7.5% (42/561) would shorten the program, and 12.7% (95/561) would extend the program. Over half of the participants (378/560, 67.5%) would recommend the program to others, while 32.5% (182/560) would not.

Almost all participants reported that the text messages were comprehensible (544/550, 98.9%). Participants were also asked if the text messages were helpful, and 384 out of 550 (69.8%) agreed that they were. A majority (327/550, 59.5%) indicated that they perceived the text messages as individually tailored to them.

**Figure 4:**
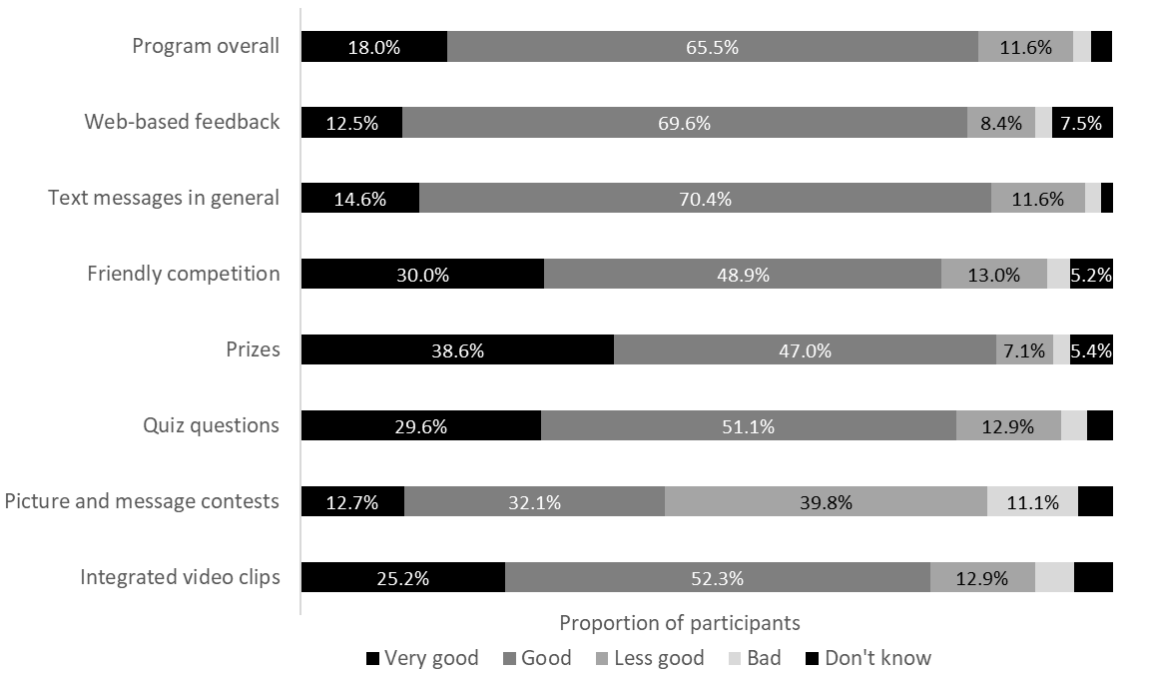
Evaluations of the program and specific program elements by program participants (n=560). Values are presented for percentages >5.0%.

Figure 4 presents additional evaluations of the program and specific program elements. The program, overall, was evaluated as *very good* or *good* by 83.6% (469/561) of the participants. Out of the specific program elements, the prizes, the text messages in general, the web-based feedback, and the quiz questions received the best evaluations, with more than 82.2% (461/561) of participants rating them as *good* or *very good*. The picture and message contests received the poorest ratings: 44.9% (252/561) of the participants rated them as *good* or *very good*.

### Initial Efficacy Based on 6-Month Follow-Up

The results of the complete-case (CC) and ITT analyses examining prevalence of problem drinking, tobacco smoking, and cannabis use are displayed in Table 3.

In the 30 days preceding the 6-month follow-up assessment, prevalence of problem drinking increased by 2.5% (from 15.2% to 17.7%) in the intervention group and by 3.4% (from 20.7% to 24.1%) in the control group, relative to that observed at baseline. This group effect was significant in the CC analysis (odds ratio [OR] 0.64, 95% CI 0.44-0.91; *P*=.01) but not in the ITT analysis (OR 0.71, 95% CI 0.49-1.03; *P*=.07). The prevalence of tobacco smoking also showed a steeper increase for controls (from 15.1% to 18.5%) compared to those who received the intervention (from 12.1% to 14.5%) from baseline to the 6-month follow-up, but this effect was not significant, neither in the ITT analysis (OR 0.83, 95% CI 0.50-1.36; *P*=.46) nor in the CC analysis (OR 0.82, 95% CI 0.46-1.43; *P*=.48). No significant group effect was observed for the pre-post difference in prevalence of cannabis smoking (+2.3% vs +1.3%; *P*_ITT_=.21; *P*_CC_=.60).

Results for continuous outcomes are summarized in Table 4.

**Table 3:** Intervention effects for dichotomous outcomes.

Outcome			Intervention group (n=750)							Control group (n=723)							*z*			*P* value			Odds ratio (95% CI)
			Baseline, n (%)		Follow-up, n (%)		Diff^a^, %		Baseline, n (%)			Follow-up, n (%)		Diff, %									
**Complete-case analysis**																							
	Problem drinking in past 30 days^b^	114 (15.2)		88/597 (14.7)		–0.5		150 (20.7)			149/635 (23.5)		2.8		–0.45			.01			0.64 (0.44-0.91)		
	Tobacco smoking in past 30 days^b^	91 (12.1)		75/597 (12.6)		0.5		109 (15.1)			114/635 (18.0)		2.9		–0.20			.48			0.82 (0.46-1.43)		
	Cannabis use in past 30 days^c^	106 (14.1)		79/596 (13.3)		–0.8		103 (14.2)			101/635 (15.9)		1.7		–0.11			.60			0.90 (0.60-1.34)		
**Intention-to-treat analysis**																							
	Problem drinking in past 30 days^b^	114 (15.2)		133 (17.7)		2.5		150 (20.7)			174 (24.1)		3.4		–0.34			.07			0.71 (0.49-1.03)		
	Tobacco smoking in past 30 days^b^	91 (12.1)		109 (14.5)		2.4		109 (15.1)			134 (18.5)		3.4		–0.19			.46			0.83 (0.50-1.36)		
	Cannabis use in past 30 days^c^	106 (14.1)		123 (16.4)		2.3		103 (14.2)			112 (15.5)		1.3		0.21			.21			1.24 (0.89-1.73)		

^a^Diff: difference.

^b^Based on generalized linear mixed models with a random effect for school classes, group as fixed factor, follow-up scores as outcomes, and baseline scores, lockdown experience, immigration background, school type, perceived stress, and problematic alcohol use at baseline as covariates.

^c^Based on generalized linear model with the follow-up scores as outcomes, group as predictor, and the baseline score, lockdown experience, immigration background, school type, perceived stress, and problematic alcohol use at baseline as covariates.

Quantity of alcohol consumed per month decreased by 0.6 standard drinks in the intervention group and increased by 0.7 standard drinks in the control group (*P*_ITT_=.03; *P*_CC_=.06) from baseline to the follow-up assessment. Further, a significant group effect was observed for pre-post differences in cigarette smoking (–1.7 cigarettes per month in the intervention group and +5.0 cigarettes per month in the control group; *P*_ITT_=.01; *P*_CC_=.07) and reported stress (–0.2 in the intervention group and no change in the control group; *P*_ITT_=.02; *P*_CC_=.03). No significant group effect was observed for frequency of cannabis smoking (+0.01 days in the intervention group and +0.39 days in the control group; *P*_ITT_=.053; *P*_CC_=.02). Pre-post differences in well-being (+4.7 in the intervention group and +3.3 in the control group; *P*_ITT_=.16; *P*_CC_=.24) and social skills (+0.5 in the intervention group and +0.3 in the control group; *P*_ITT_=.07; *P*_CC_=.10) also did not differ significantly between groups.

**Table 4:** Intervention effects for continuous outcomes.

Outcome		Intervention group (n=750)				Control group (n=723)				*t* test (*df*)		*P* value		Effect size *d* (95% CI)
		Baseline	Follow-up	Diff^a^, mean	Baseline		Follow-up	Diff, mean						
**Complete-case analysis, mean (SD)**														
	Quantity of alcohol use in past 30 days^b^	5.9 (16.3)	4.7 (11.9)	–1.2	7.5 (16.4)		8.1 (17.1)	0.6	–1.64 (1224)		.06		–0.05 (–0.16 to 0.06)	
	Quantity of cigarettes smoked in past 30 days^b^	5.3 (41.8)	3.3 (20.7)	–2.0	7.9 (47.8)		12.5 (69.9)	4.6	–5.01 (1223)		.07		–0.11 (–0.22 to 0.006)	
	Cannabis smoking days in past 30 days^b^	0.8 (3.4)	0.7 (2.9)	–0.9	0.8 (3.5)		1.2 (4.5)	0.4	–0.45 (1209)		.02		–0.15 (–0.27 to –0.04)	
	Perceived stress score^c^ in past 30 days^d^	2.9 (0.9)	2.8 (0.9)	–0.1	3.0 (0.9)		3.0 (1.0)	0	–0.19 (1209)		.03		–0.13 (–0.25 to –0.02)	
	Well-being score (WHO-5^e^)^d^	52.9 (17.3)	56.9 (16.8)	4.0	51.6 (17.3)		54.3 (18.0)	2.7	1.64 (1211)		.24		0.05 (–0.06 to 0.17)	
	Social skills score (ICQ-10^f^)^b^	14.9 (2.2)	15.4 (2.1)	0.5	14.9 (2.2)		15.2 (2.2)	0.3	0.20 (1184)		.10		0.08 (–0.03 to 0.19)	
**Intention-to-treat-analysis, mean (SD)**														
	Quantity of alcohol use in past 30 days^b^	5.9 (16.3)	5.3 (13.1)	–0.6	7.5 (16.4)		8.1 (16.8)	0.7	–1.74 (1465)		.03		–0.08 (–0.18 to 0.02)	
	Quantity of cigarettes smoked in past 30 days^b^	5.3 (41.8)	3.6 (21.1)	–1.7	7.9 (47.8)		12.9 (68.6)	5.0	–6.66 (1466)		.01		–0.13 (–0.23 to –0.03)	
	Cannabis use days in past 30 days^b^	0.8 (3.4)	0.8 (2.9)	0.0	0.8 (3.5)		1.2 (4.6)	0.4	–0.33 (1466)		.053		–0.12 (–0.22 to –0.01)	
	Perceived stress score in past 30 days^d^	2.9 (0.9)	2.7 (0.9)	–0.2	3.0 (0.9)		3.0 (1.0)	0	–0.21 (1473)		.02		–0.15 (–0.25 to –0.05)	
	Well-being score (WHO-5)^d^	52.9 (17.3)	57.6 (17.1)	4.7	51.6 (17.3)		54.9 (18.6)	3.3	2.04 (1473)		.16		0.07 (–0.04 to 0.17)	
	Social skills score (ICQ-10)^b^	14.9 (2.2)	15.4 (2.1)	0.5	14.9 (2.2)		15.2 (2.2)	0.3	0.20 (1465)		.07		0.08 (–0.02 to 0.18)	

^a^Diff: difference.

^b^Based on linear models with the change scores from baseline to follow-up as outcomes, group as predictor, and lockdown experience, immigration background, school type, perceived stress, and problematic alcohol use at baseline as covariates.

^c^Perceived stress scores range from 1 (never) to 5 (all the time).

^d^Based on linear mixed models with a random effect for school classes, group as fixed factor, change scores from baseline to follow-up as outcomes, and lockdown experience, immigration background, school type, perceived stress, and problematic alcohol use at baseline as covariates.

^e^WHO-5: 5-item World Health Organization Well-Being Index; final scores range from 0 (worst imaginable well-being) to 100 (best imaginable well-being).

^f^ICQ-10: 10-item Interpersonal Competence Questionnaire; final scores range from 5 (always poor/unable to handle social situations) to 20 (always good/able to handle social situations).

## Discussion

### Principal Findings

This study tested the appropriateness and initial effectiveness of *SmartCoach*, a mobile phone–based life-skills training program for substance use prevention in a sample of proactively recruited secondary school students in Switzerland. Three main findings were revealed: (1) 4 out of 5 secondary school students (84%) participated in the study, showing a high interest in this interventional approach; (2) overall program use and engagement was good; and (3) initial results on program efficacy showed a significant intervention effect for some of the considered outcomes, including quantity of alcohol consumed per month, quantity of cigarettes smoked per month, and reported stress.

The proactive invitation for program and study participation in secondary and upper secondary schools, in combination with the offer of a low-threshold mobile phone–based intervention, permitted us to reach 4 out of 5 adolescents for participation in the *SmartCoach* program and/or the associated study. Given the program duration of 22 weeks and that program participants needed to indicate their mobile phone number, this high participation rate of 84% is remarkable. Compared to the related *ready4life* program for life-skills training among vocational school students, the participation rate was similar, with 82% of students present within the school classes that could be recruited [26]. In contrast, substance-specific mobile phone–based programs, conducted in the same setting and using similar recruitment procedures, achieved slightly lower participation rates, with 50% to 75% of smokers participating in comparable programs to support smoking cessation [45-47] and around 75% in comparable programs for the prevention of problem drinking [48,49]. This preference for general life-skills training programs compared to substance-related programs might reflect the higher attractiveness of life skills–related topics, like stress management or social-skills training, but might also be associated with stigma and prejudice about substance-related disorders [50,51].

Concerning program use and engagement, the overall results were positive, with the majority of students (95%) remaining registered for the program for the total duration of 6 months and, on average, with program participants responding to half (mean of 23.6 out of 50) of the prompted activities. Program evaluations underlined its appropriateness for the target group of secondary school students, with the majority rating the program as helpful and individually tailored. However, 10% failed to engage in any of the 50 program activities, and another 20% showed low engagement, with less than 10 program interactions. Based on these findings, there is clearly room for improvement in terms of active program engagement, particularly concerning the picture and message contests, which received the poorest ratings among all program elements. The poor rating for this highly interactive element might be due to the limitations of mobile phone text messaging to receive and send pictures, which could be implemented more elegantly within a chat-based native smartphone app; however, direct comparisons of coaching programs based on SMS text messaging and smartphone apps concerning engagement and efficacy are still pending [52]. Compared to other text messaging–based prevention programs for adolescents, program engagement with *SmartCoach* was similar: the mean number of activities carried out by participants in the *ready4life* life-skills training program among vocational school students was 15.5 out of 39 possible activities [26]; within a smoking cessation program for vocational and upper secondary school students, participants answered a mean of 6.6 (SD 3.5) out of 11 text message prompts [53].

Further measures to increase program engagement based on the recommendations from a recent review [54] might be customizable features to provide a tailored experience and promote a sense of agency. For the *SmartCoach* program, this could include more flexibility concerning timing and extent of the intervention (eg, by the provision of fixed content at certain points in time and optional content, which the user can request flexibly). Furthermore, the provision of the right type of support at the right time by adapting to an individual’s changing internal and contextual state, as conceptualized in just-in-time adaptive interventions (JITAIs) [55], has the potential to increase program engagement and effectiveness. Although JITAIs are typically provided via smartphone apps, a recently published study demonstrated that a text messaging–based just-in-time planning intervention was effective in reducing alcohol use among adolescents [56].

The results concerning the initial effectiveness of this program based on 6-month follow-up data are promising, with three of nine outcomes of the ITT analyses showing beneficial developments of statistical significance (ie, stress, quantity of alcohol use, and quantity of tobacco use; *P*<.05) and another three outcomes (ie, problem drinking prevalence, cannabis use days, and social skills) showing beneficial developments of borderline significance (*P*<.10). As the metric measures of substance use are more sensitive to change than the binary prevalence measures, which present the main outcomes of this study and provided the basis for the power calculations, these initial results are not conclusive for the effects of the primary outcomes of this study.

### Limitations

The main limitations of this study are as follows:

1. Power calculations were based on the 18-month follow-up assessment [29]; therefore, all results concerning efficacy of the program should be considered as preliminary.
2. All data relied on self-report and the associated possibility that results may have been influenced by social desirability and a potential recall bias. Measures used to avoid under- or overreporting of substance use included assurance of confidentiality and anonymous assessments conducted via online survey and without personal contact, which may have increased the reliability of self-reported data.
3. Cluster randomization according to school class did not result in a balancing of all baseline characteristics.
4. There was selective attrition in the intervention group for persons with higher tobacco use and problem drinking at baseline. Although multiple imputations were used to compensate for this imbalance as much as possible, it would be interesting to investigate the reasons for this selective attrition. It is possible that the program reinforced cognitive dissonance and, associated with this, created a reactance toward the program. For future programs, this would mean that content should be chosen very carefully in this respect.
5. Some of the follow-up assessments were conducted during the lockdown restrictions due to the COVID-19 pandemic. This might have affected the generalizability of the results; however, this potential effect was addressed by the inclusion of a corresponding dummy covariate within all outcome analyses.
6. The results could not be generalized to secondary and upper secondary schools in Switzerland, as we recruited a convenience sample of school classes willing to participate in the study. However, the comparison of substance use prevalence rates among a representative sample of 15-year-old students in Switzerland [6] and the baseline characteristics of the study sample did not reveal major deviations. The 30-day point prevalence rates for tobacco smoking were 14% in this study and 16% in the representative survey. For cannabis use, 30-day point prevalence was also 14% in this study and 11% in the survey. Concerning alcohol, the figures are not directly comparable, with 18% of the study sample showing problem drinking in the previous 30 days according to the AUDIT-C [33] and 25% practicing binge drinking in the representative sample [6].

### Conclusions and Outlook

This is the first study that tested the appropriateness and efficacy of a mobile phone–delivered life-skills training program for substance use prevention among adolescents within a controlled trial. Our results suggest that this program, which delivers individualized messages and interactive activities integrated within a friendly competition, is both appropriate and promising in its effectiveness. Given that the program could be presented and introduced by research workers to students within one school lesson, it could be easily and economically implemented.

Our initial results indicate that the program might be effective in both preventing or reducing substance use and fostering life skills, such as coping with stress. However, data from the final 18-month follow-up will provide more robust results, including regarding potential moderators and mediators of program efficacy. Concerning moderators, it would be of particular interest to examine whether individuals with higher levels of substance use could also benefit from life-skills training programs. It would also be of particular interest to test which of the life skills addressed and successfully modified might prevent or decrease substance use.

## Appendix

Multimedia Appendix 1 CONSORT-eHEALTH checklist (V 1.6.2).

## Abbreviations

AUDIT-C: Alcohol Use Disorders Identification Test–Consumption Items
CC: complete case
GLMM: generalized linear mixed model
HBSC: Health Behaviour in School-aged Children
ICC: intraclass correlation
ICQ-10: 10-item Interpersonal Competence Questionnaire
ITT: intention to treat
JITAI: just-in-time adaptive intervention
lme4: linear mixed-effects 4
LMM: linear mixed model
mice: multivariate imputation by chained equations
OR: odds ratio
WHO-5: 5-item World Health Organization Well-Being-Index
