# Supplementary material for: A Mobile Phone–Based Life-Skills Training Program for Substance Use Prevention Among Adolescents: Cluster-Randomized Controlled Trial
Source: JMIR Mhealth Uhealth. 2021 Jul 13;9(7):e26951. doi: 10.2196/26951 (PMC8317035; doi:10.2196/26951)
Supplement: Multimedia Appendix 1 [file mhealth_v9i7e26951_app1.pdf]

CONSORT-EHEALTH Checklist V1.6.2 Report	Manuscript Number	26951
**(based on CONSORT-EHEALTH V1.6), available at [http://tinyurl.com/consort-ehealth-v1-6].**		
**Date completed**		
7/9/2021 3:39:52		
**by**		
Severin Haug		
A Mobile Phone–Based Life-Skills Training Program for Substance Use Prevention Among Adolescents: Cluster-Randomized Controlled Trial		
TITLE		
**1a-i) Identify the mode of delivery in the title**		
"A Mobile Phone–Based Life-Skills Training Program..."		
**1a-ii) Non-web-based components or important co-interventions in title**		
**1a-iii) Primary condition or target group in the title**		
"...Training Program for Substance Use Prevention Among Adolescents"		
ABSTRACT		
**1b-i) Key features/functionalities/components of the intervention and comparator in the METHODS section of the ABSTRACT**		
The efficacy of the intervention was tested in comparison to an assessment-only control group. The automated intervention program SmartCoach included online feedback and individually tailored text messages provided over 22 weeks. The contents were based on social cognitive theory and addressed self-management skills, social skills, and substance use resistance skills.		
**1b-ii) Level of human involvement in the METHODS section of the ABSTRACT**		
**1b-iii) Open vs. closed, web-based (self-assessment) vs. face-to-face assessments in the METHODS section of the ABSTRACT**		
**1b-iv) RESULTS section in abstract must contain use data**		
**1b-v) CONCLUSIONS/DISCUSSION in abstract for negative trials**		
INTRODUCTION		
**2a-i) Problem and the type of system/solution**		
"Life-skills intervention programs to prevent substance use [12-14] primarily combine training in self-management skills (eg, adapting to stress, emotional self-regulation, and goal setting), social skills (eg, assertiveness and communication skills), and skills facilitating the resistance to substance use (eg, opposing peer pressure to drink alcohol, identifying and resisting media influences that promote cigarette smoking, and correcting normative misperceptions of substance use). In spite of the fact that these life-skills training programs were compelling at preventing the onset [8,14,15] of using an explicit substance or at reducing problematic substance use [9], their implementation and dispersal in schools present genuine difficulties [16]: teachers or other professionals need time, training, knowledge, and skills to prepare and administer such programs [17]. Digital interventions have great potential to overcome the above-mentioned obstacles that hinder successful program implementation and larger-scale dissemination of life-skills training in schools. These programs have a large reach at low cost and offer the ability to deliver uniquely personalized content automatically, which can be accessed anytime and anywhere. Furthermore, digital interventions might be more appealing for adolescents because they can better ensure privacy and tailor contents to their needs."		
**2a-ii) Scientific background, rationale: What is known about the (type of) system**		
"A promising way of delivering preventive services, besides conventional personal computers, is to do so remotely by using mobile technologies. Almost all (99%) adolescents between the ages of 12 and 19 years in Switzerland, as in most other developed countries, own a mobile phone. Compared to services that can only be accessed at particular times or places, they provide a targeted and confidential means of intervention delivery [22]. Mobile phone–based interventions can provide almost constant support to users, in comparison to interventions that can only be accessed at specific times or locations, and they provide a discrete and confidential means of intervention delivery [23]. Mobile phone text messaging, in particular, is a suitable means of delivering individually tailored messages via mobile phones. This interactive service allows cost-effective, instantaneous, direct delivery of messages to individuals. Recent reviews underline the potential efficacy of text messaging–based interventions to reduce alcohol and tobacco use for different at-risk target groups, including adolescents and young adults [24,25]."		
**Does your paper address CONSORT subitem 2b?**		
"Our main hypothesis concerning the final follow-up at month 18 is that the individually tailored intervention program will be more effective than assessment only in preventing the onset and escalation of problematic alcohol and tobacco use. This study presents (1) the results on appropriateness (acceptance, use, and evaluation of duration, intensity, tailoring, helpfulness, comprehensibility, etc) of this program as well as (2) initial results on its efficacy considering 6-month follow-up assessments of this controlled trial."		
METHODS		
**3a) CONSORT: Description of trial design (such as parallel, factorial) including allocation ratio**		
"The efficacy of the intervention was tested in comparison to an assessment-only control group, considering data from the first follow-up assessment after 6 months."<br>"To avoid spillover effects within school classes, we conducted a cluster-randomized controlled trial using a school class as a randomization unit. Due to the heterogeneity of students in the different secondary schools, we used a separate randomization list for each school (ie, stratified randomization). Furthermore, to approximate equality of sample sizes in the study groups, we used block randomization with computer-generated randomly permuted blocks of 4 cases [28]."		
**3b) CONSORT: Important changes to methods after trial commencement (such as eligibility criteria), with reasons**		
There were no important changes to methods after trial commencement.		
**3b-i) Bug fixes, Downtimes, Content Changes**		
**4a) CONSORT: Eligibility criteria for participants**		
"Students were invited to participate in the study if they met the following criteria: (1) were a minimum age of 14 years, (2) owned a mobile phone, and (3) provided parental informed consent if they were under 15 years of age. Informed consent was obtained online from all study participants."		
**4a-i) Computer / Internet literacy**		
**4a-ii) Open vs. closed, web-based vs. face-to-face assessments:**		

"We tested the intervention program in secondary and upper secondary school students, typically aged between 14 and 17 years. Secondary schools in the German-speaking part of<br>Switzerland were invited to participate in the study by cooperating regional centers for addiction prevention. Employees of these centers arranged 60-minute information sessions in participating secondary school classes during regular school lessons reserved for health education."		
**4a-iii) Information giving during recruitment**		
**4b) CONSORT: Settings and locations where the data were collected**		
"We tested the intervention program in secondary and upper secondary school students, typically aged between 14 and 17 years. Secondary schools in the German-speaking part of<br>Switzerland were invited to participate in the study by cooperating regional centers for addiction prevention. Employees of these centers arranged 60-minute information sessions in participating secondary school classes during regular school lessons reserved for health education."<br>"Informed consent was obtained online from all study participants. Subsequently,<br>they were invited to choose a username, provide their mobile phone number, and fill in the baseline assessment directly on their mobile phone."<br>"Follow-up assessments in both study groups were conducted using a similar procedure: participants were invited to the online follow-up assessments via SMS text messaging, which included a link to the follow-up survey. Nonresponders were additionally addressed via computer-assisted telephone interviews conducted by research assistants."		
**4b-i) Report if outcomes were (self-)assessed through online questionnaires**		
"Informed consent was obtained online from all study participants. Subsequently,<br>they were invited to choose a username, provide their mobile phone number, and fill in the baseline assessment directly on their mobile phone."<br>"Follow-up assessments in both study groups were conducted using a similar procedure: participants were invited to the online follow-up assessments via SMS text messaging, which included a link to the follow-up survey. Nonresponders were additionally addressed via computer-assisted telephone interviews conducted by research assistants."		
**4b-ii) Report how institutional affiliations are displayed**		
**5) CONSORT: Describe the interventions for each group with sufficient details to allow replication, including how and when they were actually administered**		
**5-i) Mention names, credential, affiliations of the developers, sponsors, and owners**		
**5-ii) Describe the history/development process**		
**5-iii) Revisions and updating**		
**5-iv) Quality assurance methods**		
**5-v) Ensure replicability by publishing the source code, and/or providing screenshots/screen-capture video, and/or providing flowcharts of the algorithms used**		
**5-vi) Digital preservation**		
**5-vii) Access**		
"We tested the intervention program in secondary and upper secondary school students, typically aged between 14 and 17 years. Secondary schools in the German-speaking part of<br>Switzerland were invited to participate in the study by cooperating regional centers for addiction prevention.Employees of these centers arranged 60-minute information sessions in participating secondary school classes during regular school lessons reserved for health education. These information sessions were led by junior scientists from the Swiss Research Institute for Public Health and Addiction, who were experienced in work with young people, experienced in the provision of preventive interventions, and trained on the study and the program to be delivered."		
**5-viii) Mode of delivery, features/functionalities/components of the intervention and comparator, and the theoretical framework**		
"The intervention elements of the program were based on social cognitive theory [20,21]. The key concepts of this theory, which were addressed within SmartCoach, were (1) outcome expectations, (2) self-efficacy, (3) observational learning, (4) facilitation, and (5) self-regulation."<br>"Individually tailored web-based feedback was provided to study participants of the intervention group immediately after completion of the online baseline assessment within the school classes. It comprised seven screens, including textual and graphical feedback on stress in general, individual level of stress in various domains, individual applied and suggested coping strategies, as well as individual level of social skills. Instruments for the assessment of stress and coping strategies were derived from the Juvenir 4.0 study, a national survey on stress in adolescents with more than 1500 participants [32]. Data from this survey were also used to provide age- and gender-specific feedback on individual stress level."<br>"For a period of 22 weeks, program participants received between two and four individualized text messages per week on their mobile phone. These messages were generated and sent by the fully automated system. Within the first 7 weeks, the messages<br>focused on self-management skills (eg, coping with stress, emotional self-regulation, or management of feelings of anger and frustration). In weeks 8 to 17, the messages focused on social skills (eg, making requests, refusing unreasonable requests, and meeting new people). In weeks 18 to 22, the text messages focused on substance use resistance skills (eg, recognizing and resisting media influences, correcting normative misperceptions of substance use, or understanding the associations of self-management skills and social skills with substance use). The messages were tailored according to the individual data from the baseline assessment and were based on text messaging assessments during the program runtime (eg, on substance use or on the individual's emotional state). Several interactive features, such as quiz questions, tasks to create individually tailored if-then behavior plans based on implementation intentions, and message contests, were implemented within the program. Due to the wide dissemination of smartphones among adolescents [22], several messages also included hyperlinks to audio files (eg, audio testimonials and motivational podcasts) as well as to thematically appropriate video clips, pictures, and related websites."		
**5-ix) Describe use parameters**		
**5-x) Clarify the level of human involvement**		
**5-xi) Report any prompts/reminders used**		
"For a period of 22 weeks, program participants received between two and four individualized text messages per week on their mobile phone."		
**5-xii) Describe any co-interventions (incl. training/support)**		
"Within the first half of the information sessions in the school classes, the junior scientists raised awareness about the importance of life skills to effectively cope with the demands<br>and challenges of everyday life. For this purpose, they used video sequences demonstrating typical stressors and demands for this age group (eg, search for an apprenticeship, exam stress, and peer pressure for substance use) and different strategies to cope with them. The importance of emotional regulation skills and social skills to effectively cope with these stressors were discussed based on case vignettes. Subsequently, the students were informed about, and invited to participate in, a study testing innovative channels for the provision of life-skills training."		
**6a) CONSORT: Completely defined pre-specified primary and secondary outcome measures, including how and when they were assessed**		

"Baseline and follow-up assessments included the following: 1. Problem drinking and alcohol use in the preceding 30 days, assessed by the short form of the Alcohol Use Disorders Identification Test–Consumption Items (AUDIT-C) [33]. This test is comprised of three items: (1) frequency of alcohol consumption, (2) quantity of alcohol consumption, and (3) binge drinking. Pictures were used to illustrate the quantity of a standard drink, which corresponded to 12 g to 14 g of pure alcohol. Based on a validation study of a large German sample, a cutoff score of ≥5 was used [34]. 2. The 30-day point prevalence rate for smoking abstinence (ie, not having smoked a puff within the past 30 days according to the criteria of the Society for Nicotine and Tobacco Research [35]). 3. Quantity of cigarettes smoked in the preceding 30 days, assessing by the number of smoking days and the typical number of cigarettes smoked per smoking day. 4. Cannabis use in the preceding 30 days, assessed by an item of the HBSC (Health Behaviour in School-aged Children) study [36] addressing the number of cannabis consumption days.5. Perceived stress, assessed by a single item from the Swiss Juvenir study [32]—"How often have you had the feeling of being overstressed or overwhelmed in the last month?"—with answer options ranging from 1 (never) to 5 (all the time). 6. Well-being, assessed by the 5-item World Health Organization Well-Being Index (WHO-5) [37], with the final score ranging from 0, representing the worst imaginable well-being, to 100, representing the best imaginable well-being.7. Social skills, assessed by the brief version of the 10-item Interpersonal Competence Questionnaire (ICQ-10) [38] addressing the following domains: (1) initiation of relationships, (2) negative assertion, (3) disclosure of personal information, (4) emotional support, and (5) conflict management. The primary outcomes, according to the study protocol [29], are (1) prevalence of problem drinking in the preceding 30 days according to the AUDIT-C and (2) prevalence of cigarette smoking in the preceding 30 days (ie, having smoked at least a puff, according to the criteria of the Society for Nicotine and Tobacco Research [35]). Secondary outcomes were (1) prevalence of cannabis use in the preceding 30 days (ie, having used cannabis at least once), (2) quantity of alcohol use in the preceding 30 days, (3) quantity of cigarettes smoked in the previous 30 days, (4) frequency of cannabis use in the preceding 30 days, (5) perceived stress, (6) well-being, and (7) social skills."<br><br>"Students were invited to participate in the study if they met the following criteria: (1) were a minimum age of 14 years, (2) owned a mobile phone, and (3) provided parental informed consent if they were under 15 years of age. Informed consent was obtained online from all study participants. Subsequently, they were invited to choose a username, provide their mobile phone number, and fill in the baseline assessment directly on their mobile phone."<br><br>"Follow-up assessments in both study groups were conducted using a similar procedure: participants were invited to the online follow-up assessments via SMS text messaging, which included a link to the follow-up survey. Nonresponders were additionally addressed via computer-assisted telephone interviews conducted by research assistants."		
**6a-i) Online questionnaires: describe if they were validated for online use and apply CHERRIES items to describe how the questionnaires were designed/deployed**		
**6a-ii) Describe whether and how "use" (including intensity of use/dosage) was defined/measured/monitored**		
**6a-iii) Describe whether, how, and when qualitative feedback from participants was obtained**		
**6b) CONSORT: Any changes to trial outcomes after the trial commenced, with reasons**		
"We tested the intervention program in secondary and upper secondary school students, typically aged between 14 and 17 years. Secondary schools in the German-speaking part of Switzerland were invited to participate in the study by cooperating regional centers for addiction prevention. Employees of these centers arranged 60-minute information sessions in participating secondary school classes during regular school lessons reserved for health education."<br>"Informed consent was obtained online from all study participants. Subsequently, they were invited to choose a username, provide their mobile phone number, and fill in the baseline assessment directly on their mobile phone."<br>"Follow-up assessments in both study groups were conducted using a similar procedure: participants were invited to the online follow-up assessments via SMS text messaging, which included a link to the follow-up survey. Nonresponders were additionally addressed via computer-assisted telephone interviews conducted by research assistants."		
**7a) CONSORT: How sample size was determined**		
**7a-i) Describe whether and how expected attrition was taken into account when calculating the sample size**		
**7b) CONSORT: When applicable, explanation of any interim analyses and stopping guidelines**		
"Baseline and follow-up assessments included the following: 1. Problem drinking and alcohol use in the preceding 30 days, assessed by the short form of the Alcohol Use Disorders Identification Test–Consumption Items (AUDIT-C) [33]. This test is comprised of three items: (1) frequency of alcohol consumption, (2) quantity of alcohol consumption, and (3) binge drinking. Pictures were used to illustrate the quantity of a standard drink, which corresponded to 12 g to 14 g of pure alcohol. Based on a validation study of a large German sample, a cutoff score of ≥5 was used [34]. 2. The 30-day point prevalence rate for smoking abstinence (ie, not having smoked a puff within the past 30 days according to the criteria of the Society for Nicotine and Tobacco Research [35]). 3. Quantity of cigarettes smoked in the preceding 30 days, assessing by the number of smoking days and the typical number of cigarettes smoked per smoking day. 4. Cannabis use in the preceding 30 days, assessed by an item of the HBSC (Health Behaviour in School-aged Children) study [36] addressing the number of cannabis consumption days.5. Perceived stress, assessed by a single item from the Swiss Juvenir study [32]—"How often have you had the feeling of being overstressed or overwhelmed in the last month?"—with answer options ranging from 1 (never) to 5 (all the time). 6. Well-being, assessed by the 5-item World Health Organization Well-Being Index (WHO-5) [37], with the final score ranging from 0, representing the worst imaginable well-being, to 100, representing the best imaginable well-being.7. Social skills, assessed by the brief version of the 10-item Interpersonal Competence Questionnaire (ICQ-10) [38] addressing the following domains: (1) initiation of relationships, (2) negative assertion, (3) disclosure of personal information, (4) emotional support, and (5) conflict management. The primary outcomes, according to the study protocol [29], are (1) prevalence of problem drinking in the preceding 30 days according to the AUDIT-C and (2) prevalence of cigarette smoking in the preceding 30 days (ie, having smoked at least a puff, according to the criteria of the Society for Nicotine and Tobacco Research [35]). Secondary outcomes were (1) prevalence of cannabis use in the preceding 30 days (ie, having used cannabis at least once), (2) quantity of alcohol use in the preceding 30 days, (3) quantity of cigarettes smoked in the previous 30 days, (4) frequency of cannabis use in the preceding 30 days, (5) perceived stress, (6) well-being, and (7) social skills."<br><br>"Students were invited to participate in the study if they met the following criteria: (1) were a minimum age of 14 years, (2) owned a mobile phone, and (3) provided parental informed consent if they were under 15 years of age. Informed consent was obtained online from all study participants. Subsequently, they were invited to choose a username, provide their mobile phone number, and fill in the baseline assessment directly on their mobile phone."<br><br>"Follow-up assessments in both study groups were conducted using a similar procedure: participants were invited to the online follow-up assessments via SMS text messaging, which included a link to the follow-up survey. Nonresponders were additionally addressed via computer-assisted telephone interviews conducted by research assistants."		
**8a) CONSORT: Method used to generate the random allocation sequence**		
"Due to the heterogeneity of students in the different secondary schools, we used a separate randomization list for each school (ie, stratified randomization). Furthermore, to approximate equality of sample sizes in the study groups, we used block randomization with computer-generated randomly permuted blocks of 4 cases [28]."		
**8b) CONSORT: Type of randomisation; details of any restriction (such as blocking and block size)**		
"Due to the heterogeneity of students in the different secondary schools, we used a separate randomization list for each school (ie, stratified randomization). Furthermore, to approximate equality of sample sizes in the study groups, we used block randomization with computer-generated randomly permuted blocks of 4 cases [28]."		
**9) CONSORT: Mechanism used to implement the random allocation sequence (such as sequentially numbered containers), describing any steps taken to conceal the sequence until interventions were assigned**		
"Due to the heterogeneity of students in the different secondary schools, we used a separate randomization list for each school (ie, stratified randomization). Furthermore, to approximate equality of sample sizes in the study groups, we used block randomization with computer-generated randomly permuted blocks of 4 cases [28]."		
**10) CONSORT: Who generated the random allocation sequence, who enrolled participants, and who assigned participants to interventions**		
"Due to the heterogeneity of students in the different secondary schools, we used a separate randomization list for each school (ie, stratified randomization). Furthermore, to approximate equality of sample sizes in the study groups, we used block randomization with computer-generated randomly permuted blocks of 4 cases [28]."<br>"Junior scientists supervising the baseline assessment were blinded to the group allocation of school classes. In addition, group allocation was not revealed to participants until they had provided their informed consent, username, mobile phone number, and baseline data. Furthermore, the research assistants who performed the computer-assisted follow-up assessments for primary and secondary outcomes were blinded to the group allocation."		
**11a) CONSORT: Blinding - If done, who was blinded after assignment to interventions (for example, participants, care providers, those assessing outcomes) and how**		
**11a-i) Specify who was blinded, and who wasn't**		
"Junior scientists supervising the baseline assessment were blinded to the group allocation of school classes. In addition, group allocation was not revealed to participants until they had provided their informed consent, username, mobile phone number, and baseline data. Furthermore, the research assistants who performed the computer-assisted follow-up assessments for primary and secondary outcomes were blinded to the group allocation."		
**11a-ii) Discuss e.g., whether participants knew which intervention was the "intervention of interest" and which one was the "comparator"**		

**11b) CONSORT: If relevant, description of the similarity of interventions**		
The intervention and assessment only control group were not intended to be similar.		
**12a) CONSORT: Statistical methods used to compare groups for primary and secondary outcomes**		
"We analyzed data according to the intention-to-treat (ITT) principle. For the ITT analyses, we used multiple imputation procedures as described elsewhere [39]. We imputed for each group separately to preserve homogeneity within the groups and potential interventional effects. Overall predictors of missing data at follow-up were gender, immigration background, education, and number of students within a school class. Differential predictors of missing data at follow-up were problem drinking, tobacco smoking, and use of the program. Thus, these predictors were part of all imputation models for the study's primary and secondary outcomes. The remaining study outcome predictors were variables that correlated at least weakly with these (r>0.20). Binary variables were imputed using logistic regression, categorical variables using multinomial logit models, and continuous variables using predictive mean matching. We examined 50 data sets and no systematic bias in convergence was revealed; thus, the final inferences were derived from this solution. Next, we calculated the intraclass correlation (ICC) for primary and secondary outcomes. In our study, the ICC determines the extent to which study outcomes vary across classrooms. If an ICC is close to 0, standard regressions provide unbiased coefficients, whereas an ICC higher than 0 indicates that hierarchical models are needed to avoid a type I statistical error. In previous studies, ICCs between 0.05 and 0.10 were considered negligible [40,41]. However, it is an open debate as to how well the ICC performs depending on the underlying data [42]. Thus, we opted for a conservative approach and conducted linear mixed models (LMMs) and generalized linear mixed models (GLMMs) where the ICC was higher than 5%, and logistic or linear regressions where the ICC was below 5%. Within LMMs and GLMMs, we modeled a random intercept for school class, while predictors and covariates were identical to logistic or linear regressions. Analyses of binary outcomes focused on follow-up values. Independent variables included baseline values for the binary variables of interest, group as a predictor, and variables for which baseline differences were observed as covariates. Analyses of continuous outcomes included change in score from baseline to follow-up as the dependent variable. Independent variables included group as a predictor and variables for which baseline differences were observed. We included in all models a covariate that modeled the possible effect of the lockdown measures undertaken in Switzerland between February 28 and June 22, 2020, because of the COVID-19 pandemic. During this period, several parts of students' lives were affected (eg, schools and/or bars were closed), which may have had an effect on our outcomes. The results from the imputed data set were cross-checked with the nonimputed data set. Results with a type I error rate of P<.05 on two-sided tests were considered statistically significant. Analyses were performed using SPSS, version 25 (IBM Corp), and R, version 3.6.1 (The R Foundation). Multiple imputation was conducted with the mice (multivariate imputation by chained equations) package in R [43], and LMM and GLMM were conducted with the lme4 (linear mixed-effects 4) package in R [44]."		
**12a-i) Imputation techniques to deal with attrition / missing values**		
"We analyzed data according to the intention-to-treat (ITT) principle. For the ITT analyses, we used multiple imputation procedures as described elsewhere [39]. We imputed for each group separately to preserve homogeneity within the groups and potential interventional effects. Overall predictors of missing data at follow-up were gender, immigration background, education, and number of students within a school class. Differential predictors of missing data at follow-up were problem drinking, tobacco smoking, and use of the program. Thus, these predictors were part of all imputation models for the study's primary and secondary outcomes. The remaining study outcome predictors were variables that correlated at least weakly with these (r>0.20). Binary variables were imputed using logistic regression, categorical variables using multinomial logit models, and continuous variables using predictive mean matching. We examined 50 data sets and no systematic bias in convergence was revealed; thus, the final inferences were derived from this solution."		
**12b) CONSORT: Methods for additional analyses, such as subgroup analyses and adjusted analyses**		
"Within LMMs and GLMMs, we modeled a random intercept for school class, while predictors and covariates were identical to logistic or linear regressions. Analyses of binary outcomes focused on follow-up values. Independent variables included baseline values for the binary variables of interest, group as a predictor, and variables for which baseline differences were observed as covariates. Analyses of continuous outcomes included change in score from baseline to follow-up as the dependent variable. Independent variables included group as a predictor and variables for which baseline differences were observed."		
RESULTS		
**13a) CONSORT: For each group, the numbers of participants who were randomly assigned, received intended treatment, and were analysed for the primary outcome**		
"Figure 3 depicts participants' progression through the trial. At the online screening assessment, 1759 students were present in 89 classes. Of these, 1623 (92.3%) students received parental approval to participate, and 1473 (83.7%) students ultimately participated in the study. A total of 44 classes containing 750 students in total were randomly assigned to the intervention group, and 45 classes containing 723 students in total were assigned to the control group. Follow-up assessments at 6 months were completed by 597 out of 750 (79.6%) participants in the intervention group and 636 out of 723 (88.0%) participants in the control group."		
**13b) CONSORT: For each group, losses and exclusions after randomisation, together with reasons**		
Participants`progression through the trial and reasons for losses are depicted in Figure 3.		
**13b-i) Attrition diagram**		
**14a) CONSORT: Dates defining the periods of recruitment and follow-up**		
"Study participants were recruited between March 2019 and March 2020. The 6-month follow-up assessments were conducted between August 2019 and September 2020."		
**14a-i) Indicate if critical "secular events" fell into the study period**		
**14b) CONSORT: Why the trial ended or was stopped (early)**		
The trial was ended regularly after the target sample size was reached.		
**15) CONSORT: A table showing baseline demographic and clinical characteristics for each group**		
Table 2 presents baseline characteristics of the study sample for each group.		
**15-i) Report demographics associated with digital divide issues**		
Table 2 presents baseline characteristics of the study sample for each group.		
**16a) CONSORT: For each group, number of participants (denominator) included in each analysis and whether the analysis was by original assigned groups**		
**16-i) Report multiple "denominators" and provide definitions**		
Tables 3 and 4 report on intervention effects and on number of participants used for each comparison.		
**16-ii) Primary analysis should be intent-to-treat**		
**17a) CONSORT: For each primary and secondary outcome, results for each group, and the estimated effect size and its precision (such as 95% confidence interval)**		
Tables 3 and 4 report on intervention effects, estimated effect sizes and their precision.		
**17a-i) Presentation of process outcomes such as metrics of use and intensity of use**		
**17b) CONSORT: For binary outcomes, presentation of both absolute and relative effect sizes is recommended**		
Absolute and relative effects sizes for binary outcomes are reported in Table 3.		
**18) CONSORT: Results of any other analyses performed, including subgroup analyses and adjusted analyses, distinguishing pre-specified from exploratory**		
We did not perform subgroup analyses or further adjusted analyses.		
**18-i) Subgroup analysis of comparing only users**		
**19) CONSORT: All important harms or unintended effects in each group**		
Harms or futher unintended effects beyond those measured in the outcomes were not assessed.		
**19-i) Include privacy breaches, technical problems**		
**19-ii) Include qualitative feedback from participants or observations from staff/researchers**		
DISCUSSION		
**20) CONSORT: Trial limitations, addressing sources of potential bias, imprecision, multiplicity of analyses**		
**20-i) Typical limitations in ehealth trials**		

"The main limitations of this study are as follows:<br>1. Power calculations were based on the 18-month follow-up assessment [29]; therefore, all results concerning efficacy of the program should be considered as preliminary.<br>2. All data relied on self-report and the associated possibility that results may have been influenced by social desirability and a potential recall bias. Measures used to avoid underor<br>overreporting of substance use included assurance of confidentiality and anonymous assessments conducted via online survey and without personal contact, which may have increased the reliability of self-reported data.<br>3. Cluster randomization according to school class did not result in a balancing of all aseline characteristics.<br>4. There was selective attrition in the intervention group for persons with higher tobacco use and problem drinking at baseline. Although multiple imputations were used to compensate for this imbalance as much as possible, it would be interesting to investigate the reasons for this selective attrition. It is possible that the program reinforced cognitive dissonance and, associated with this, created a reactance toward the program. For future programs, this would mean that content should be chosen very carefully in this respect.<br>5. Some of the follow-up assessments were conducted during the lockdown restrictions due to the COVID-19 pandemic. This might have affected the generalizability of the results; however, this potential effect was addressed by the inclusion of a corresponding dummy covariate within all outcome analyses.<br>6. The results could not be generalized to secondary and upper secondary schools in Switzerland, as we recruited a convenience sample of school classes willing to participate<br>in the study."		
**21) CONSORT: Generalisability (external validity, applicability) of the trial findings**		
**21-i) Generalizability to other populations**		
**21-ii) Discuss if there were elements in the RCT that would be different in a routine application setting**		
**22) CONSORT: Interpretation consistent with results, balancing benefits and harms, and considering other relevant evidence**		
**22-i) Restate study questions and summarize the answers suggested by the data, starting with primary outcomes and process outcomes (use)**		
"This study tested the appropriateness and initial effectiveness of SmartCoach, a mobile phone–based life-skills training program for substance use prevention in a sample of proactively recruited secondary school students in Switzerland. Three main findings were revealed: (1) 4 out of 5 secondary school students (84%) participated in the study, showing a high interest in this interventional approach; (2) overall program use and engagement was good; and (3) initial results on program efficacy showed a significant intervention effect for some of the considered outcomes, including quantity of alcohol consumed per month, quantity of cigarettes smoked per month, and reported stress."		
**22-ii) Highlight unanswered new questions, suggest future research**		
Other information		
**23) CONSORT: Registration number and name of trial registry**		
"Trial Registration: ISRCTN Registry ISRCTN41347061; https://doi.org/10.1186/ISRCTN41347061"		
**24) CONSORT: Where the full trial protocol can be accessed, if available**		
"Their implementation within the SmartCoach program is described in more detail within the<br>study protocol [29]."		
**25) CONSORT: Sources of funding and other support (such as supply of drugs), role of funders**		
"Funding for this project was provided by the Swiss National Science Foundation (grant 10001C_179222/1). The funding institution did not influence the design and conduct of the study; the management, analysis, or interpretation of data; or the preparation, review, or approval of the manuscript."		
**X26-i) Comment on ethics committee approval**		
**x26-ii) Outline informed consent procedures**		
**X26-iii) Safety and security procedures**		
**X27-i) State the relation of the study team towards the system being evaluated**		
